# Method for quantification of porcine type I interferon activity using luminescence, by direct and indirect means

**DOI:** 10.1186/s12896-022-00743-9

**Published:** 2022-03-29

**Authors:** Michael Puckette, J. Barrera, M. Schwarz, M. Rasmussen

**Affiliations:** 1 Plum Island Animal Disease Center, U. S. Department of Homeland Security Science and Technology Directorate, P.O. Box 848, Greenport, NY 11944 USA; 2 Plum Island Animal Disease Center, Leidos, Inc., P.O. Box 848, Greenport, NY 11944 USA; 3 Oak Ridge Institute for Science and Education, Plum Island Animal Disease Center Research Participation Program, P.O. Box 848, Greenport, NY 11944 USA

**Keywords:** *Gaussia* luciferase, Interferon α, Interferon β, Fusion protein, VSV, FMDV, Luciferase, Assay, Anti-viral

## Abstract

**Background:**

Type I interferons are widely used in research applications and as biotherapeutics. Current assays used to measure interferon concentrations, such as plaque reduction assays and ELISA, are expensive, technically challenging, and may take days to provide results. We sought to develop a robust and rapid assay to determine interferon concentrations produced from transiently transfected cell cultures.

**Method:**

Indirect quantification of recombinant interferon was evaluated using a novel bi-cistronic construct encoding the Foot-and-mouth disease virus 2A translational interrupter sequence to yield equimolar expression of *Gaussia princeps* luciferase and porcine interferon α. Direct quantification was evaluated by expression of a novel fusion protein comprised of *Gaussia princeps* luciferase and porcine type I interferon. Plasmids encoding constructs are transiently transfected into cell cultures and supernatant harvested for testing of luminescence, ELISA determined concentration, and anti-viral activity against vesicular stomatitis virus.

**Results:**

Bi-cistronic constructs, utilized for indirect quantification, demonstrate both luciferase activity and anti-viral activity. Fusion proteins, utilized for direct quantification, retained secretion and luminescence however only the interferon α fusion protein had antiviral activity comparable to wildtype porcine interferon α. A strong linear correlation was observed between dilution and luminescence for all compounds over a dynamic range of concentrations.

**Conclusion:**

The correlation of antiviral and luciferase activities demonstrated the utility of this approach, both direct and indirect, to rapidly determine recombinant interferon concentrations. Concentration can be determined over a more dynamic concentration range than available ELISA based assays using this methodology.

**Supplementary Information:**

The online version contains supplementary material available at 10.1186/s12896-022-00743-9.

## Background

Type I interferons, IFNα and IFNβ, are used as biotherapeutics to treat a number of medical conditions including, leukemia, melanoma, human papillomavirus, chronic hepatitis B and C, and multiple sclerosis. Porcine IFNα and IFNβ have been used to inhibit Vesicular Stomatitis Virus (VSV), Porcine Reproductive and Respiratory Syndrome Virus, and Foot-and-Mouth Disease Virus (FMDV) in livestock [1–3].

Interferons are typically quantified through antibody-capture assays, or through assays that measure anti-viral biological activity, such as plaque reduction assays [4–6]. Comparing interferon levels among samples with these assays can be problematic, especially when conducting mutational analyses that may disrupt target epitopes or interferon activity [7]. Rapid quantification of interferon levels, independent of antibody-capture or anti-viral activity, would aid interferon research and development, especially through increased screening of large sample numbers.

Previously we demonstrated that the addition of a 30 amino acid sequence comprising the FMDV translational interrupter sequence (Δ1D2A) to either the N- or C-terminus of *Gaussia* luciferase (GLuc), a naturally secreted luciferase isolated from *Gaussia princeps* [8–10], does not prevent either GLuc secretion or luminescence [11]. Furthermore, GLuc activity can be measured directly in biological samples, including blood, serum, and urine [9, 10]. The addition of the Δ1D2A sequence to a GLuc 8990 mutant (SGLuc), which enhances luciferase output in the presence of cell lysis buffers [11, 12], also has no effect on SGLuc secretion or luminescence.

**Fig. 1:**
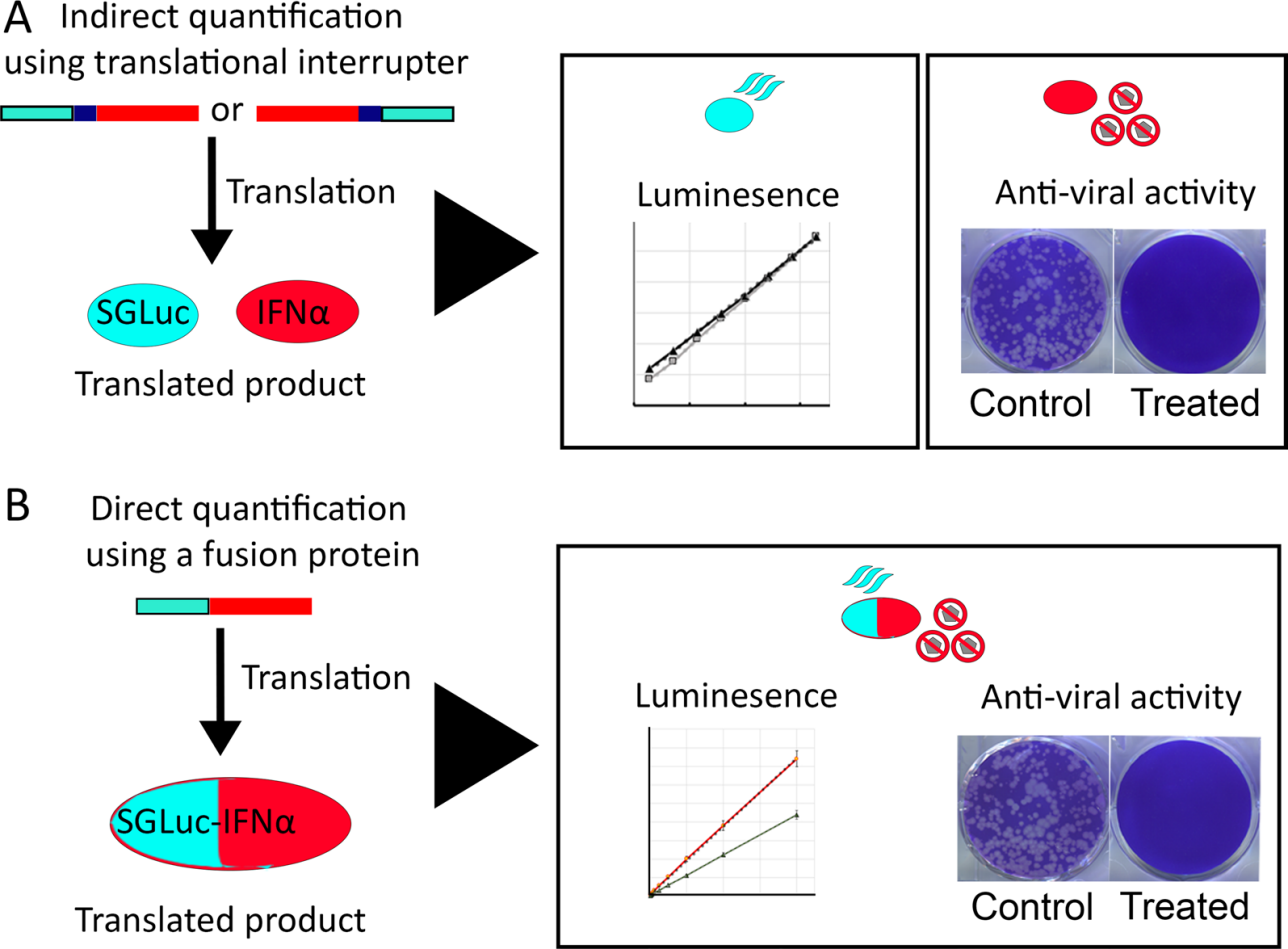
Schematic diagram of methodology utilizing luminescence to quantify IFNα. **A** Indirect quantification is achieved through the FMDV 2A translational interrupter sequence, blue, that results in expression of luciferase and IFNα as separate proteins in an equimolar ratio in cell culture media. **B** Direct quantification is achieved utilizing a fusion protein consisting of SGLuc and IFNα that is secreted into cell culture media. The SGLuc-IFNα fusion protein retains both luminescence and anti-viral activity

To determine if SGLuc- and Δ1D2A-containing constructs could be used to accurately quantify recombinant IFNα expression, we used the SGLuc-Δ1D2A and Δ1D2A-SGLucΔ1M variants to produce bicistronic single open reading frame vectors expressing SGLuc and porcine IFN proteins. These constructs represented an indirect interferon concentration assay because the luciferase and IFN proteins are separated upon translation, Fig. 1A. Supernatant from transiently transfected cell cultures are subsequently evaluated for luciferase activity, derived from SGLuc, and anti-viral activity, derived from IFNα.

Interferon fusion proteins have been used for: (1) incorporation of reporter molecules [13, 14]; (2) immunotherapy [15–18]; (3) enhancement of half-life [19–21]; (4) enhancement of activity [22], and (5) facilitation of secretion in non-mammalian systems [23]. To measure the interferon concentration directly via luciferase activity we constructed fusion proteins of SGLuc and porcine IFNα or IFNβ, identified as SGLuc-IFNα and SGLuc-IFNβ, respectively. In these SGLuc-IFN fusion proteins, the IFNα and IFNβ secretion signal peptides, normally removed during secretion by membrane-bound peptidases [24–29], were replaced with SGLuc. Because these constructs lack the Δ1D2A they remain a single expressed protein after translation which is capable of both luciferase and anti-viral activity, Fig. 1B.

This study evaluates both bicistronic and fusion protein constructs expressing SGLuc and type I interferons and utilizes luciferase activity to quantify interferon produced from transiently transfected cell lines.

## Methods

### Construction of expression plasmids

Synthetically synthesized sequences for porcine IFNα, IFNβ, SGLuc-IFNα and SGLuc-IFNβ were inserted into the pUC57kan vector (Genscript USA Inc) and subsequently cloned into a modified pTARGET™ vector (mpTarget) for mammalian expression. The sequence for IFNα was inserted into previously constructed Δ1D2A-SGLucΔ1M and SGLuc-Δ1D2A constructs for bi-cistronic vectors [11]. These constructs differ in whether the luciferase is on the N- or C- terminus of the Δ1D2A Foot-and-Mouth Disease Virus derived translational “skipping” mechanism. For the Δ1D2A-SGLucΔ1M construct the first methionine of SGLuc is also deleted. Plasmids were transformed into NEB® 5-α Competent *E. coli* (New England Biolabs) and plated on LB Agar plates with 100 μg/mL carbenicillin (Teknova, L1010). Selected colonies were grown in 4 mL of Terrific Broth with 100 μg/mL carbenicillin (Teknova, T7030) overnight at 37 °C, and plasmid purification was performed using QIAprep® Spin Miniprep kit (Qiagen, 27,106). Insertion was validated by sequence analysis using primers mpTarget-F (GACATCCACTTTGCCTTTCTCTC) and mpTarget-R (CTCATCAATGTATCTTATCATGTC). Recombinant plasmid DNAs were purified utilizing a EndoFree Plasmid Maxi kit (Qiagen, 12,362).

### Transfection of HEK293-T cells

Purified plasmid DNA was used to transfect HEK293-T cells (ATCC, CRL-3216), passage 48, at roughly 80% confluence in 6-well plates (Costar, 3516). Prior to transfection, growth media, composed of 1X DMEM, 10% fetal bovine serum, 1X antibiotic–antimycotic, and 1X non-essential amino acids, was removed, and cells were rinsed with 1X dPBS (Gibco™, 14,190,250). Fresh media, 1 mL, was applied and transfections were performed using 4 μg of plasmid DNA and Lipofectamine™ 2000 (Thermo Fisher, 11,668,027). Cells were incubated overnight at 37 °C in a 5% CO_2_ incubator. Media from transfected cells was harvested and stored in aliquots of 200 μL at -70 °C.

After removal of media, cells were washed with 1 mL of 1X dPBS (Gibco™, 14,190,250) and lysed by adding 1 mL of Mammalian Protein Extraction Reagent (M-PER™; Thermo Fisher, 78,501) with repeated pipetting. Lysates were stored at − 70 °C.

### Quantification of luminescence

Luminescence was assayed by injecting 100 μL of 50 μM water soluble coelenterazine (Nanolight, 3031–10) into 100 μL of harvested media diluted with fresh cell culture media. Luminescence was quantified from samples in 96-well white LUMITRAC™ 200 polystyrene microplates (Greiner Bio-one, 781075) analyzed in a 96-well Veritas™ microplate luminometer (Turner Biosystems) with an integration time of 0.5 s. Data is represented in relative luciferase units per half second (RLUs/0.5 s).

### Western blotting of cell culture supernatant

Western blotting of cell culture supernatant was performed utilizing 60 μL of sample mixed with 30 μL of 4× NuPage LDS Sample Buffer (Invitrogen, NP0007), heated at 97C for 10 min and 15 μL loaded into wells of 10-well NuPage 4–12% Bis–Tris gel (Invitrogen, NP0321Box). Gels were electrophoresed in 1× MES buffer (Invitrogen, NP0002) at 200 V for 35 min followed by transfer onto PVDF Pre-cut blotting membranes (Invitrogen, LC2002) utilizing the iBlot2 system (Invitrogen).

Membranes were incubated in a blocking buffer of 5% milk for 1 h at room temperature, then washed three times with 1X PBS-T (EMD Millipore, 524653-1EA) for 5 min each. Primary antibodies, polyclonal Antisera GLuc (Nanolight Technology, 401P), anti-IFNα (pbl Assay Science, 27100-1 Lot: 5795), and anti-IFNβ (ATCC, ab136385 Lot: GR142674-6), were added at 1:1000 dilution and incubated for 1 h at room temperature. Membranes were washed three times with 1× PBS-T for 5 min after the primary antibody incubation, and 1:500 dilutions of the secondary antibodies, goat anti-mouse-HRP (LGC Seracare, 5220-0338) or goat anti-rabbit-HRP (LGC Seracare, 5220-0335) applied to membranes for 1 h at room temperature. After three washes of 1× PBS-T membranes were incubated using the SIGMAFAST 3,3′-diaminobenzidine tablets (Sigma, D4293-50SET) as suggested by the manufacturer.

### Cytopathic effect inhibition assay

Interferon induced inhibition of infection by VSV was used to evaluate interferon biological activity due to well-characterized acute VSV sensitivity to interferon [6, 30–32]. Cytopathic effect inhibition assays (CEI) were performed on samples as described previously [33]. Bovine derived MDBK cells (ATCC, CCL-22), passage 134, were infected with VSV-New Jersey (VSV-NJ) at a 0.0028 multiplicity of infection (MOI). Antiviral activity was reported as Interferon Antiviral Activity, the absence of cytopathogenic effect in 50% of tested wells, per 100 μL (IFNAA_50_/100 μL) or as the number of samples containing plaques.

### Determination of IFN and SGLuc-IFN interferon concentrations by ELISA

Concentrations of porcine IFNα and SGLuc-IFNα samples were determined using a Porcine IFNα ELISA kit (Millipore Sigma, RAB1131-1KT), and a Porcine Interferon Beta ELISA kit (Novateinbio, NB-E50024) was used for IFNβ and SGLuc-IFNβ samples. Absorbance was recorded at OD_450 nm_ using an ELx 808 ultra microplate reader (BIO-TEK Instruments). Samples of IFNα and SGLuc-IFNα were assayed with two separate lots of the IFNα ELISA kit (Millipore Sigma, RAB1131-1KT) using at least three dilutions of each sample. Results were averaged to calculate the initial sample concentration.

### VSV plaque assays

Plaque assays were performed using MDBK cells (ATCC, CCL-22), passage 134, plated on 6-well plates (Costar, 3516). Samples were tested in triplicate and results were averaged. MDBK cells, grown to full confluence, were treated with select dilutions of porcine IFNα, porcine IFNβ, SGLuc-IFNα, or SGLuc-IFNβ media. Commercially available porcine IFNα and porcine IFNβ were used as positive controls. After cells were incubated at 37 °C overnight with 5% CO_2_, media was removed, and cells were gently washed with dPBS. Fresh media was applied, and cells were infected with VSV-NJ at a MOI of 0.0002 and incubated overnight at 37 °C with 5% CO_2_. After incubation, media was removed, and cells were stained with 500 µL of 0.5% crystal violet in 20% Methanol to aid in visualizing plaques.

### FMDV plaque assays

Plaque assays using FMDV were performed on FMDV permissive LFBK-αvβ6 cells, a porcine derived cell line transformed to stably express the bovine αvβ6 integrin, [34, 35], cultured in 6-well plates (Costar, 3516) to full confluence, treated with SGLuc-IFNα at appropriate dilution, and incubated overnight at 37 °C with 5% CO_2_. After incubation, media was removed and 1 mL of supplemented DMEM (+ 2% FBS, + 1% Anti-Anti, + 1% L-Glut, + 1% NEAA) media was applied gently to avoid disrupting the cell monolayer. The supplemented media was reduced to 100 µL followed by adding of 100 PFUs of FMDV serotype O1 Manisa to each well. After 1 h of incubation at 37 °C in 5% CO_2_, 2 mL of an overlay (50% gum, 50% 2× MEM supplemented with 2% FBS, 1% anti-anti, 1% NEAA, 1% L-Glut) was added to each well, and plates were incubated overnight. The next day plates were stained with 1 mL of crystal violet and plaques were counted.

## Results and discussion

### Indirect assay of IFNα levels using luminescence

**Fig. 2:**
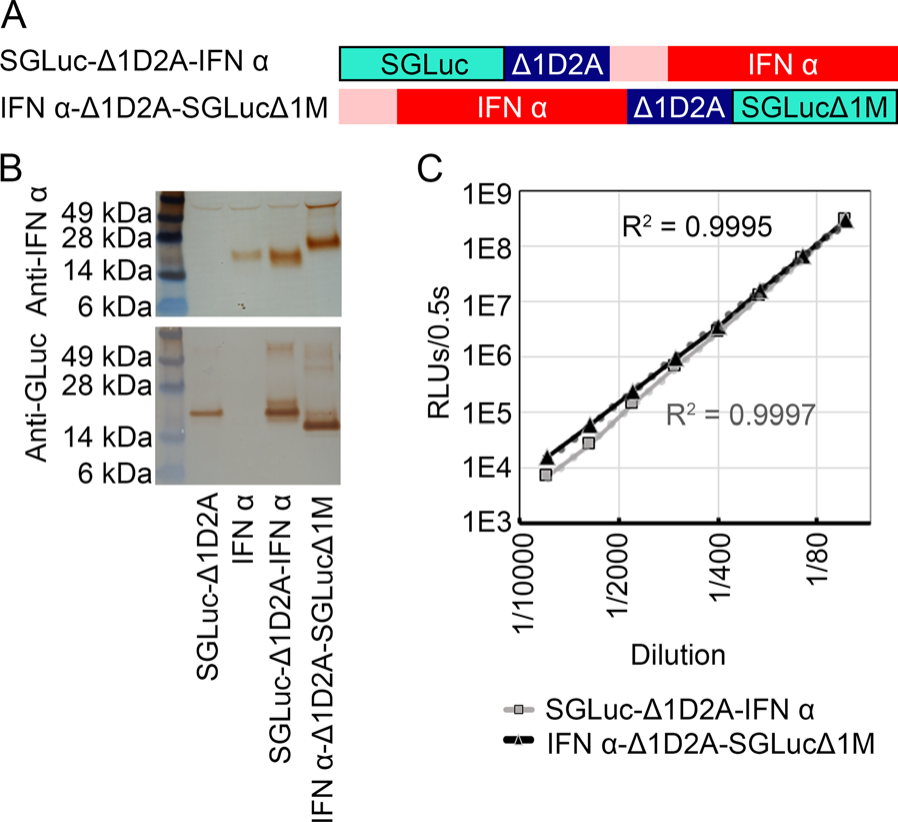
**A** Diagrams of bi-cistronic SGLuc-Δ1D2A-IFNα and IFNα-Δ1D2A-SGLucΔ1M constructs evaluated for correlation of luciferase and antiviral activities. Pink box represents the IFNα secretion peptide sequence. **B** Western blots using anti-porcine IFNα and anti-GLuc antibodies demonstrated protein banding patterns with expected sizes and confirmed secretion into cell culture media. Blots were cropped to focus on results and representative of N = 2 replicates; uncropped full-length blots are included in Additional file 1: Fig. S1. **C** Luminescence of serially diluted media, N = 5, samples from cultures producing SGLuc-Δ1D2A-IFNα and IFNα-Δ1D2A-SGLuc Δ1M. Sample dilutions range from 1/50 to 1/6400. Dotted lines represent the line of best fit

Cell culture media containing recombinant IFNα and SGLuc expressed from bi-cistronic vectors SGLuc-Δ1D2A-IFNα or IFNα-Δ1D2A-SGLucΔ1M, Fig. 2A, was assayed for luminescence and antiviral activity and compared to porcine IFNα expressed alone. Western blotting with anti-IFNα and anti-GLuc antibodies confirmed expression, secretion, and separation of the individual luciferase and interferon proteins from bi-cistronic vectors, Fig. 2B. Media from positive cell cultures was serially diluted, and luciferase activity was quantified for both IFNα-Δ1D2A-SGLuc Δ1M and SGLuc-Δ1D2A-IFNα samples. For samples produced by both constructs, a strong correlation (R^2^ > 0.99) was seen between relative luciferase units per half second (RLUs/0.5 s) and dilutions, Fig. 2C.

To further characterize the parameters of ELISA determined concentration, antiviral activity, and luciferase activity for the bi-cistronic constructs, we analyzed three independent batches produced by transfection with different concentrations of plasmid DNA, Table 1. The correlations among the three parameters was ≥ 0.96 for samples produced with the IFNα-Δ1D2A-SGLucΔ1M construct. Concentrations for the three batches of SGLuc-Δ1D2A-IFNα were not divergent with ELISA determined concentrations for all samples being within obtained standard deviations, complicating any attempt at validating correlations among tested parameters.

**Table 1:** Activities of SGLuc-Δ1D2A-IFNα and IFNα-Δ1D2A-SGLuc Δ1M

	Concentration measured by ELISA (μg/mL; ± SD)	Luminescence (RLUs/0.5 s)		CEI Assay IFNAA_50_ (log_10_)/100 µl	
		Average	SD	24 h	36 h
IFNα-Δ1D2A-SGLuc Δ1M					
Batch 1	5.58 ± 1.39	2.99 × 10^10^	1.14 × 10^9^	4.85	4.78
Batch 2	6.93 ± 1.97	3.23 × 10^10^	1.14 × 10^9^	5.00	4.93
Batch 3	2.27 ± 0.84	1.31 × 10^10^	5.81 × 10^8^	4.55	4.55
SGLuc-Δ1D2A-IFNα					
Batch 1	9.89 ± 1.15	2.61 × 10^10^	5.78 × 10^8^	5.15	5.08
Batch 2	9.56 ± 1.20	2.13 × 10^10^	6.05 × 10^8^	5.30	5.15
Batch 3	11.68 ± 3.98	2.40 × 10^10^	7.30 × 10^8^	5.30	5.15

Concentration, luminescence, and anti-VSV activity for three batches of SGLuc-Δ1D2A-IFNα and IFNα-Δ1D2A-SGLuc Δ1M. Concentration was determined by porcine IFNα ELISA, luminescence by reactivity with coelenterazine (N = 3 replicates per sample), and anti-VSV activity by CEI (N = 4 replicates per sample). RLUs/0.5 s, relative luciferase units per half second; CEI, Cytopathic effect inhibition assay; IFNAA_50_/100 μL, Interferon Antiviral Activity per 100 μL

### Expression of SGLuc and type I interferon fusion proteins

**Fig. 3:**
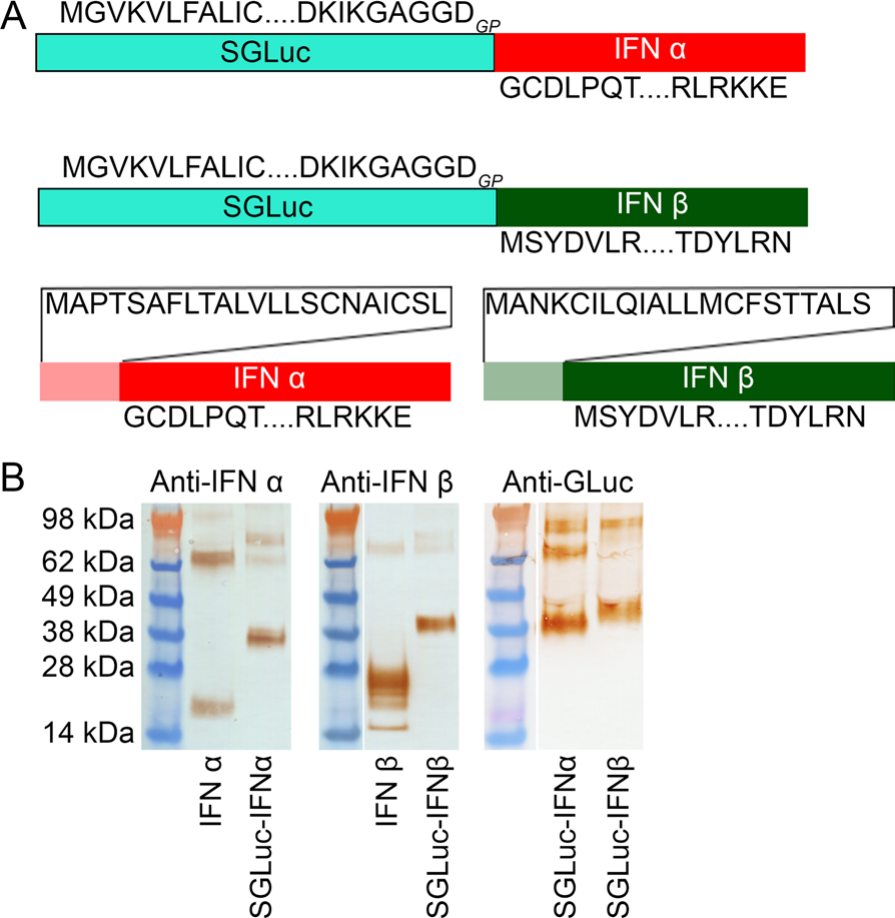
**A** Diagrams of mono-cistronic constructs expressing SGLuc-IFNα, SGLuc-IFNβ, IFNα, and IFNβ. In SGLuc-IFN constructs, the native secretion domains of porcine IFNα and IFNβ, pale red and pale green respectively, are replaced with the amino acid sequence for SGLuc. **B** Western blots of media from transfected cell cultures expressing native porcine IFN and chimeric SGLuc-IFNs using anti-IFNα, anti-IFNβ, and anti-GLuc antibodies demonstrate expression and secretion into cell culture media. Blots are cropped to focus on results and representative of N = 3 replicates; uncropped full-length blots are included in Additional file 1: Fig. S2

Fusion proteins of SGLuc and IFN, Fig. 3A, were developed to measure interferon activity using luminescence as a proxy. For these fusion proteins, the first 22 amino acids of IFNα and IFNβ, encoding the native IFN secretion domains, were removed so secretion would be dependent upon SGLuc, Fig. 3A.

Expression and secretion of both fusion proteins, SGLuc-IFNα and SGLuc-IFNβ, was compared to native IFNα and IFNβ respectively. IFNα constructs produced bands correlating in size to monomers and probable aggregates, while IFNβ constructs produced a series of bands ranging in size from 15 to 28 kDa, suggesting post-translational modifications such as glycosylation, Fig. 3B. SGLuc-IFNα produced three bands at approximately 38 kDa, 70 kDa, and 80 kDa, Fig. 3B. The 38 kDa band is consistent with the predicted size of a monomer of SGLuc-IFNα with additional bands likely IFNα aggregate. SGLuc-IFNβ produced bands roughly 40 kDa and 80 kDa in size, consistent with the predicted size of a monomer and a probable aggregate, Fig. 3B. The lack of multiple bands in fusion-protein samples, as seen with similarly expressed control IFNβ, suggested that SGLuc-IFNβ does not undergo the same degree of post-translational modification as IFNβ.

### Correlation of concentration, luciferase, and antiviral activity of SGLuc-IFN

#### Anti-viral activity of SGLuc-IFNα

**Fig. 4:**
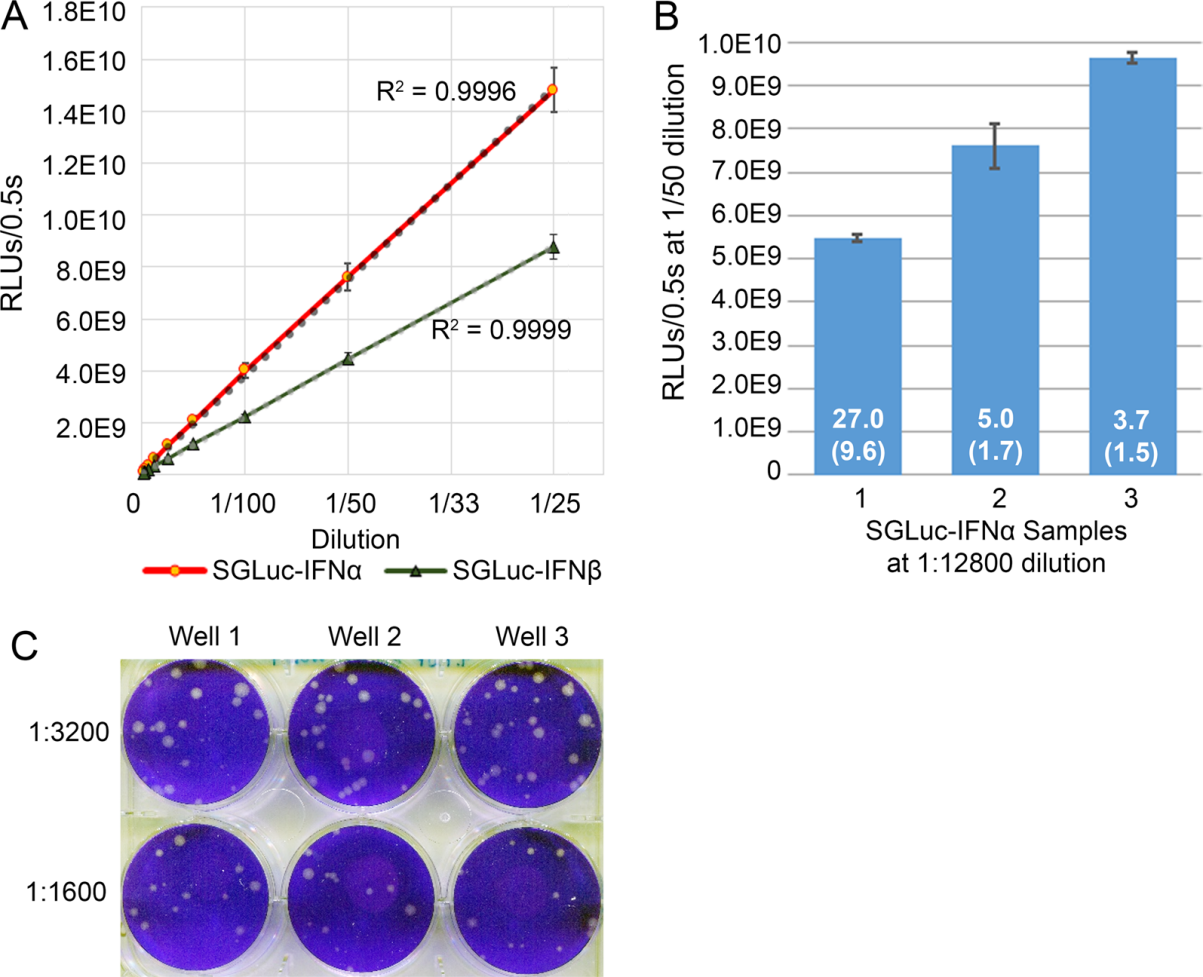
**A** Luciferase readings for serial dilutions of SGLuc-IFNα and β samples show strong linear correlation over tested dilutions of 1/50 to 1/6400. **B** Luciferase activity, N = 7 replicates per sample, in media produced from three separate transfections of SGLuc-IFNα expressing plasmid. Corresponding mean plaque numbers for each sample are within bars with standard deviation in parenthesis below; N = 3 replicates per sample. RLUs/0.5 s, relative luciferase units per half second. **C** Triplicate wells for plaque assays evaluating SGLuc-IFNα efficacy against FMDV. Tested dilutions demonstrate a reduction in plaque numbers from untreated control values of 200 to an average of 14.7 and 29.3 for dilutions of 1:1600 and 1:3200, respectively

The luminescence of media containing SGLuc-IFNα was linear over a dynamic range of dilutions, R^2^ = 0.99, Fig. 4A. To correlate luciferase activity with antiviral activity, production cells were transfected with three different concentrations of SGLuc-IFNα encoding plasmids to produce independent batches. The resulting samples were used to compare luciferase activity with anti-viral activity as determined by plaque reduction assay.

The CEI assay was used to determine dilutions at which plaques could be found, data not shown. A 1/12800 dilution was selected for all three SGLuc-IFNα batches for plaque assays, and luciferase activity was determined using a 1/50 dilution, Fig. 4B. Luminescence and the mean number of plaque forming units were found to be inversely correlated (r =  − 0.90) utilizing Pearson correlation, indicating a correlation between luminescence and the anti-viral activity of SGLuc-IFNα.

A single SGLuc-IFNα batch was used to confirm anti-viral activity against FMDV. Dilutions of 1/1600 and 1/3200 were selected based on CEI assay results, data not shown, and FMDV plaque production confirmed that the anti-viral effect of SGLuc-IFNα was not limited to VSV, Fig. 4C.

#### Anti-viral activity of SGLuc-IFNβ

The luminescence of media containing SGLuc-IFNβ was linear over a dynamic range of dilutions, R^2^ = 0.99, Fig. 4A. A 1:100 dilution of media containing SGLuc-IFNβ was required to completely inhibit VSV plaque formation in the CEI assay compared to a 1:1600 dilution of IFNβ, Table 2. The decreased potency of SGLuc-IFNβ may be due to the lack of post-translational modifications seen in Fig. 3B. Because of the reduced antiviral activity, SGLuc-IFNβ samples were not tested further.

**Table 2:** Cytopathic effect inhibition assay with IFNβ and SGLuc-IFNβ samples

Sample	No. samples with vesicular stomatitis virus plaques in diluted growth media							
	1:100	1:200	1:400	1:800	1:1600	1:3200	1:6400	1:12,800
IFNβ	0/4	0/4	0/4	0/4	0/4	2/4	4/4	4/4
SGLuc-IFNβ	0/4	4/4	4/4	4/4	4/4	4/4	4/4	4/4

For determination of anti-viral activity for IFNβ and SGLuc-IFNβ samples, four wells for each dilution were evaluated for the presence of VSV plaques at 24 h post-infection. Wells were scored positive when plaques were observed

### Comparison of SGLuc-IFNα and IFNα antiviral activity with concentrations determined by ELISA

Using Pearson correlation, we determined that there was a correlation, r =  − 0.85, between the antiviral activity and the interferon concentration in the IFNα and SGLuc-IFNα preparations, Table 3. Among the three preparations of SGLuc-IFNα alone, the antiviral activity was also correlated with concentration as determined by ELISA, r =  − 0.76. While comparisons of anti-viral activity between ELISA standardized IFNα and SGLuc-IFNα batches was not as linear as desired, this data demonstrated that fusion of SGLuc and IFNα to produce SGLuc-IFNα retained anti-viral activity when using equivalent concentrations. Future research may improve the understanding of this relationship.

**Table 3:** Comparison of anti-viral activity for different concentrations of IFNα and SGLuc-IFNα

	Source	Dilution	Concentration	PFU (± SD)
			pg/mL (± SD)	
IFNα	Batch 1	1/102400	404 ± 151	80 ± 9.0
IFNα	Batch 1	1/51200	807 ± 301	33 ± 3.2
SGLuc-IFNα	Batch 1	1/12800	738 ± 114	27 ± 9.6
SGLuc-IFNα	Batch 2	1/12800	948 ± 114	5.0 ± 1.7
SGLuc-IFNα	Batch 3	1/12800	1449 ± 214	3.7 ± 1.5

Concentration, as determined by ELISA, and SVV plaque forming units (PFU) in triplicate wells from select dilutions of IFNα and SGLuc-FNα. Three preparations of SGLuc-IFNα were diluted to 1/12,800 for comparison across batches

## Conclusion

This study sought to develop a method to rapidly determine cell culture-produced recombinant interferon concentrations through use of luminescence. We evaluated both direct and indirect means of measuring interferon activity. Direct covalent linking of SGLuc and IFNα into a single fusion-protein resulted in a high correlation between luminescence and anti-viral activity. Compared to current assays, the use of luminescence to quantify interferon concentrations enabled linear correlations across a dynamic range of concentrations, was faster, required less sample input, and was more compatible with high-throughput screening. Similar fusion proteins may be valuable research tools as fast and efficient means to analyze recombinant interferon concentrations. Further, the ability to detect GLuc activity in biological samples such as blood, serum, and urine opens the possibility of quick quantification of SGLuc-type molecules in animal models of disease or in ex vivo clinical samples.

## Supplementary Information

**Additional file 1: **Source image files for western blots.

## Data Availability

The datasets used and/or analyzed during the current study are available from the corresponding author on reasonable request. Sequences of all constructs are available in U.S. patents 10,435,695 and 10,829,770.

## Abbreviations

IFNα: Interferon α
IFNβ: Interferon β
VSV: Vesicular stomatitis virus
FMDV: Foot-and-mouth disease virus
GLuc: *Gaussia* Luciferase
SGLuc: 8990 GLuc mutant
MOI: Multiplicity of infection
CEI: Cytopathic effect inhibition assays
IFNAA_50_/100 μL: Interferon Antiviral Activity per 100 μL
