# Supplementary material for: Method for quantification of porcine type I interferon activity using luminescence, by direct and indirect means
Source: BMC Biotechnol. 2022 Mar 29;22:13. doi: 10.1186/s12896-022-00743-9 (PMC8966355; doi:10.1186/s12896-022-00743-9)
Supplement: Supplementary file 1 — Additional file 1: Source image files for western blots. [file 12896_2022_743_MOESM1_ESM.docx]

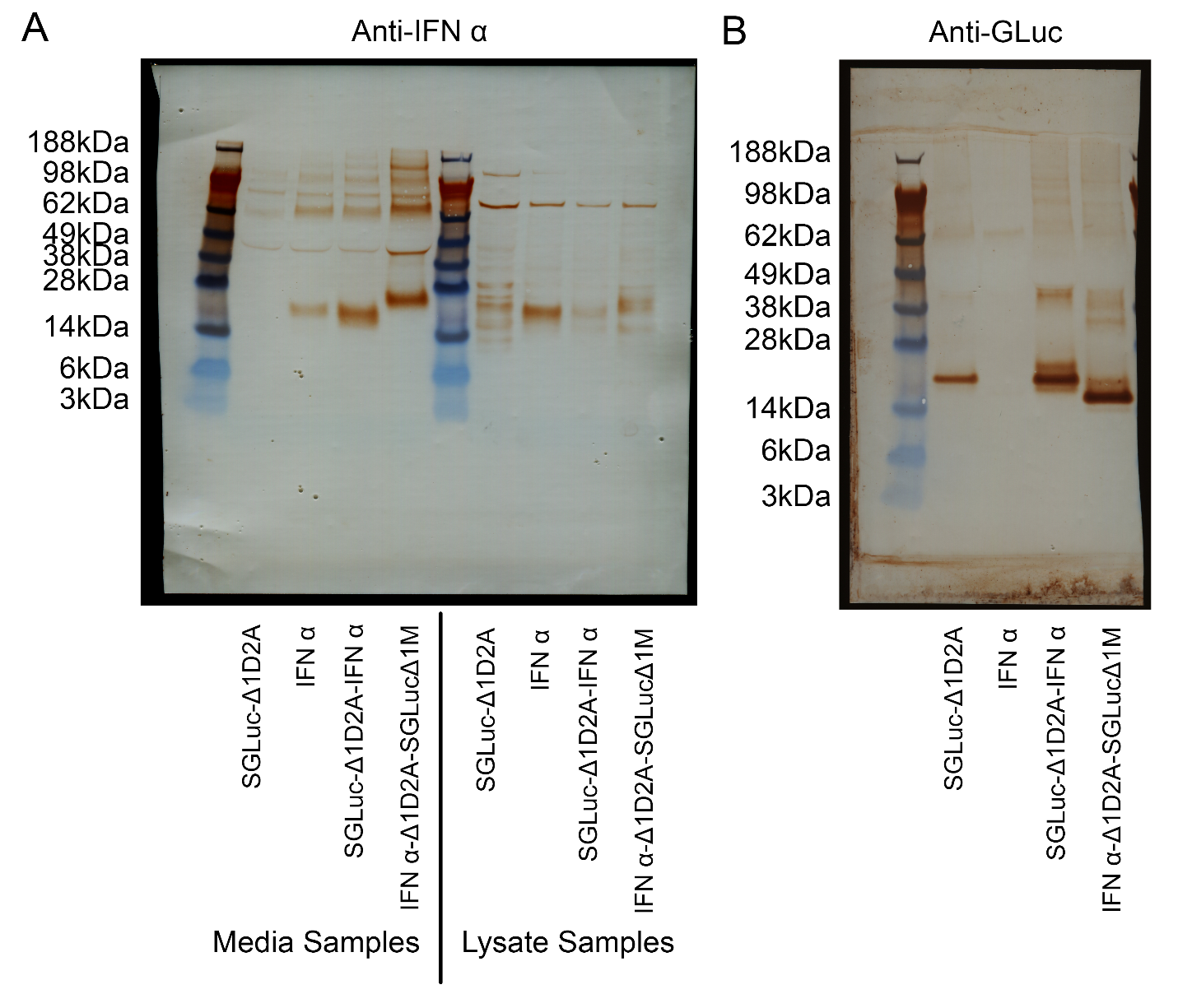


Additional file 1: Figure S1. Uncropped western blots using (A) anti-porcine IFN α and (B) anti-GLuc antibodies demonstrated protein banding patterns with expected sizes and secretion into cell culture media.


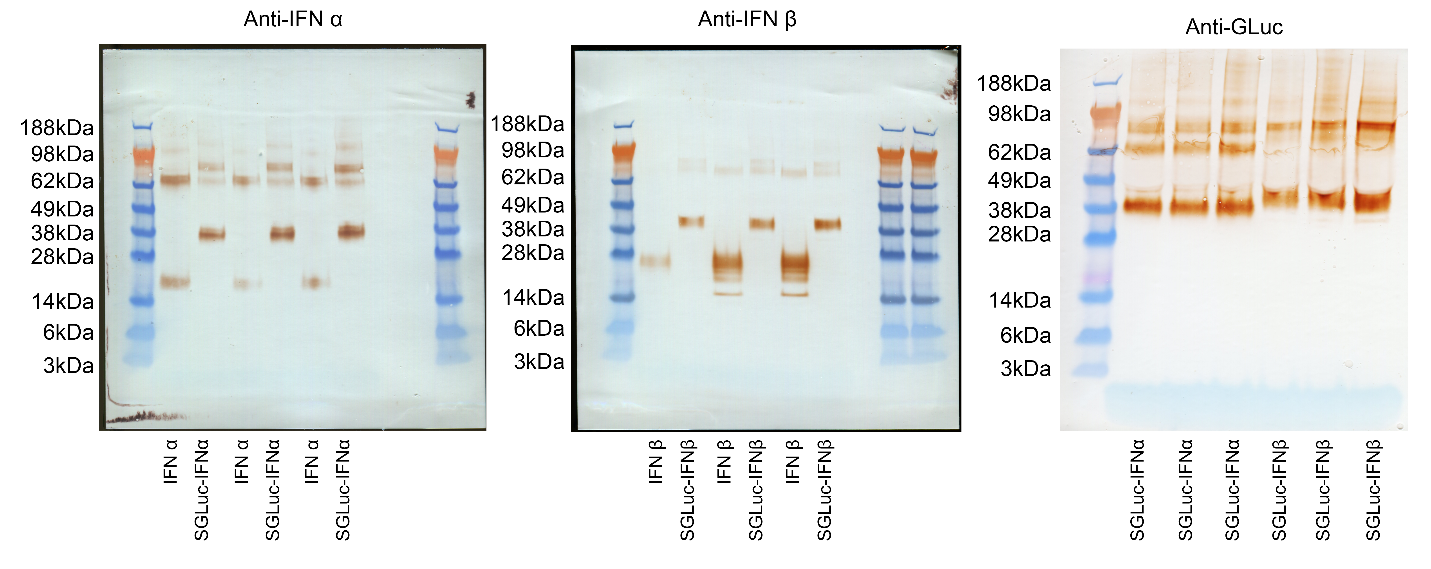


Additional file 1: Figure S2. Uncropped western blots using anti-IFN α, anti-IFN β, and anti-GLuc antibodies of media from different transfected cell cultures batches expressing native porcine IFN and chimeric SGLuc-IFNs demonstrate expression and secretion into cell culture media.
